# BRCA1/2 mutations are not a common cause of malignant melanoma in the Polish population

**DOI:** 10.1371/journal.pone.0204768

**Published:** 2018-10-04

**Authors:** Tadeusz Dębniak, Rodney J. Scott, Bohdan Górski, Bartłomiej Masojć, Andrzej Kram, Romuald Maleszka, Cezary Cybulski, Katarzyna Paszkowska-Szczur, Aniruddh Kashyap, Dawid Murawa, Karolina Malińska, Magdalena Kiedrowicz, Emilia Rogoża-Janiszewska, Helena Rudnicka, Jakub Deptuła, Paweł Domagała, Wojciech Kluźniak, Marcin R. Lener, Jan Lubiński

**Affiliations:** 1 Department of Genetics and Pathomorphology, International Hereditary Cancer Center, Pomeranian Medical University in Szczecin, Szczecin, Poland; 2 School of Biomedical Sciences and Pharmacy, Faculty of Health, University of Newcastle and the Hunter Medical Research Institute, Newcastle, New South Wales, Australia; 3 West Pomeranian Oncology Center, Szczecin, Poland; 4 Department of Skin Diseases and Venerology PUM, Police, Poland; 5 I Department of Oncological and General Surgery, Greater Poland Cancer Center, Poznań, Poland; 6 Department of Pathology, Pomeranian Medical University in Szczecin, Szczecin, Poland; CNR, ITALY

## Abstract

The association of BRCA1/2 mutations with melanoma is not completely determined; the interpretation of variants of unknown significance is also problematic. To evaluate these issues we explored the molecular basis of melanoma risk by performing whole-exome sequencing on a cohort of 96 unrelated Polish early-onset melanoma patients and targeted sequencing of BRCA1/2 genes on additional 30 melanoma patients with familial aggregation of breast and other cancers. Sequencing was performed on peripheral blood. We evaluated MutationTaster, Polyphen2, SIFT, PROVEAN algorithms, analyzed segregation with cancer disease (in both families with identified BRCA2 variants) and in one family performed LOH (based on 2 primary tumors). We found neither pathogenic mutations nor variants of unknown significance within BRCA1. We identified two BRCA2 variants of unknown significance: c.9334G>A and c.4534 C>T. Disease allele frequency was evaluated by genotyping of 1230 consecutive melanoma cases, 5000 breast cancer patients, 3500 prostate cancers and 9900 controls. Both variants were found to be absent among unselected cancer patients and healthy controls. The MutationTaster, Polyphen2 and SIFT algorithms indicate that c.9334G>A is a damaging variant. Due to lack of tumour tissue LOH analysis could not be performed for this variant. The variant segregated with the disease. The c.4534 C>T variant did not segregate with disease, there was no LOH of the variant. The c.9334G>A variant, classified as a rare variant of unknown significance, on current evidence may predisposes to cancers of the breast, prostate and melanoma. Functional studies to describe how the DNA change affects the protein function and a large multi-center study to evaluate its penetrance are required.

## Introduction

Melanoma is one of the most aggressive human malignancies. The incidence of malignant melanoma (MM) of the skin is increasing worldwide, with the most rapid increase observed in Caucasian populations [1]. In Poland the number of affected individuals has increased by more than 60% over the past eleven years and now accounts for over 2600 new diagnosed annually. The etiology of melanoma is complex, involving both genetic and environmental factors. Significant heritability of this malignancy was reported from large twin studies [2]. Family studies provide strong support for heterogeneous mechanisms involved in an inherited susceptibility to melanoma, likely as a result of polygenic factors that influence both inherited high-risk and low-risk alleles.

To date one major melanoma susceptibility gene: *CDKN2A* has been identified that is responsible for only up to 20% of melanoma cases [3]. Mutations within other MM high risk genes, such as *CDK4*, *ACD*, *CXC*, *TERT*, *TERF2IP*, *POT1* or *BAP1* are extremely rare and to date they have only been identified in a few families worldwide [3, 4, 5]. The list of intermediate- to low-penetrance risk alleles include variants in the *MC1R* [6, 7, 8], *XPD* [9], *VDR* [10], and *MITF* [11]. However, in the vast majority of MM cases the molecular background remains unclear. A possible association between melanoma and breast cancer has been reported in some studies, which suggest an independent involvement of BRCA2 in melanoma development [12]. Although the association of *BRCA*1 and *BRCA*2 mutations with breast and ovarian cancer risk is well-defined, it has not been fully explored and two significant questions remain; 1. the potential association of *BRCA1* and *BRCA2* mutations with other malignancies such as melanoma is not completely determined; and 2. the interpretation of variants of unknown significance on cancer risk is remains problematic.

To evaluate these issues we explored the molecular background of melanoma by performing whole-exome sequencing on a cohort of 96 unrelated Polish patients who were diagnosed with disease before the age of 40 yrs. All patients have previously been tested and found to be negative for any deleterious change in *CDKN2A*. Targeted sequencing of *BRCA1/2* in the 96 MM patients and an additional 30 melanoma cases with a familial aggregation of cancers that included breast cancer. The variants identified in the MM discovery phase were validated by genotyping them using a large series of 1230 consecutive MM cases and 1700 melanoma controls, 5000 consecutive breast cancer patients and 3500 prostate cancer patients and compared to 9900 controls. Prostate cancer patients were included since a small proportion of them are associated with causative germline variants in *BRCA1* or *BRCA2* [13]. For those patients who were found to carry a *BRCA1* or *BRCA2* variant segregation analysis was undertaken in their respective families.

## Materials and methods

### Study subjects

We studied three non-overlapping groups of melanoma patients. The first group included 96 unrelated patients with early-onset melanoma; the second group 30 unrelated MM patients with a familial cancer aggregation among 1^st^ and 2^nd^ degree relatives that included breast cancer—both of these groups were used for the discovery phase of the study. The third group consisted of 1230 unselected (for age, gender, cancer family history, grading or other characteristics) MM patients for the validation phase. In the validation phase we also studied a group of 5000 women with unselected breast cancer, 3500 men with unselected prostate cancer and 9900 healthy controls. Overall, patient participation rates exceeded 75%. All patients and control subjects were of European ancestry and ethnic Poles. The Polish study was approved by the ethics committee of Pomeranian Medical University (Szczecin, Poland). All study subjects provided a signed consent form for participation in the study.

### Discovery phase

In the first stage of this study we have performed whole exome sequencing in a cohort of 96 unrelated patients with early-onset (<40 yrs) melanoma from CDKN2A-negative families (65 females, mean age 30,8; range 15–40; 31 males, mean age 30,9, range 18–39). 29 out of this 96 cases were sporadic early onset melanoma patients with negative cancer family history, 38 patients were characterized by a familial aggregation of melanoma among first- or second-degree relatives, 29 were early onset melanoma patients with familial aggregation of other cancers (one or more cancers among 1^st^ or 2^nd^ degree relatives). Cases were diagnosed between 2006 and 2015 and selected from cancer registries in six Polish cities (Szczecin, Opole, Bialystok, Zielona Gora, Gorzow Wielkopolski and Poznan).

They were asked to participate at the time of diagnosis or during an outpatient visit to an oncology clinic and were selected for their age. In the second stage we performed targeted BRCA1/2 sequencing in cohort of 30 unrelated MM patients (28 females, mean age of diagnosis of 50.8 years, range 47–70; 2 males diagnosed at 47 and 70) with a familial aggregation of cancers that were not melanoma among 1^st^ and 2^nd^ degree relatives. In all these families breast cancer was one of the malignancies diagnosed among the affected relatives. Four female MM probands also developed breast cancer. Patients were diagnosed between 2003 and 2015 in Szczecin. They were asked to participate at the time of diagnosis or during an outpatient visit to an oncology clinic and were selected for their family history.

All patients had previously been screened and found not to carry *CDKN2A*-causative variants.

### Validation phase

We verified the role of any detected variants using a large case control-study of 1,230 unselected melanoma patients (760 females, mean age 53,9, range 15–92; 470 males, mean age 55, range 18–84) from Poland that came from two cohorts. The first consisted of 748 unselected MM cases (464 women, mean age 53.7 y; 284 men) diagnosed between 2002 and 2006 and identified from cancer registries in five Polish cities (Szczecin, Opole, Bialystok, Zielona Gora, and Gorzow Wielkopolski). The registries capture more than 95% of all diagnosed melanomas. The second group consisted of 482 unselected MM cases (296 females,186 males) diagnosed in Szczecin between 2010 and 2016.

The breast case series consisted of 5000 prospectively ascertained unselected female patients with invasive breast cancer (age range of 18–92 years; mean age of 53.2 years) who were diagnosed from 2008 to 2013 at 18 different hospitals in Poland. All patients diagnosed with invasive breast cancer at participating centers were eligible.

Patients were unselected for family history. The patient participation rate was 76.1%. Women with a previous contralateral breast cancer or with a current diagnosis of bilateral cancer were considered to be bilateral.

The prostate case series consisted of 3500 men with unselected prostate cancer (age range: 41–96 years; mean age: 68.8), who were diagnosed between 1999 and 2012 in 14 centers situated throughout Poland. This study was initiated in Szczecin in 1999 and was extended to include Bialystok, Olsztyn in 2002 and Opole in 2003. Other centers began recruiting between 2005 and 2008 (Koszalin, Gdansk, Lublin, Lodz, Warszawa, Wroclaw, Poznan, Rzeszow, Bydgoszcz, Zabrze). All men with prostate cancer were invited to participate. Study subjects were asked to participate at the time of diagnosis or during an outpatient visit to an oncology clinic and were unselected for age or family history. The participation rate was 86.4%.

### Controls

Melanoma control group consisted of 1696 healthy adults: 943 women (mean age, 64 years) and 753 men (mean age, 67 years) with no cancers diagnosed in their families. The healthy adults were deemed as having a negative cancer family history (first- and second-degree relatives included) after answering a questionnaire about their family's medical history which was part of a population-based study of the 1.5 million residents of West Pomerania (northwest Poland) to identify familial aggregations of malignancies among first and second degree relatives from 1.258 million residents (87%) who were registered with the West Pomeranian Regional Health Foundation.

During the interview, the goals of the study were explained, informed consent was obtained, genetic counseling was given and a blood sample was taken for DNA analysis.

Individuals affected with any malignancy or with cancers diagnosed among first- or second-degree relatives were excluded from our study control group.

The breast control group included 4,702 cancer-free women aged 18 to 94 years (mean age of 53.0 years). These controls were derived from four sources. The first subgroup consisted of 979 women from the region of Szczecin (age range of 24 to 84 years) who were chosen for this study to be matched by age and geographical region with a series of patients with incident breast cancer diagnosed in Szczecin between 1996 and 2004. These women were part of a population-based study of the 1.5 million residents of West described above. The second control series consisted of 1,707 unselected women (age range of 32–72 years) who participated in breast ultrasonography screening at 8 different centers across Poland between 2009 and 2011 and provided blood samples for DNA analysis. Women with breast cancer and women with a positive family history of breast cancer were excluded from this group. The third control group included 1,031 unselected women (age range of 20–94 years) selected at random from the computerized patient lists of family practices located in the region of Opole (south Poland). These women were invited to participate by mail and participated in 2012 and 2013. The fourth series included 985 Polish women (age range of 50–66 years) who participated in the population colonoscopy screening program for colorectal cancer between 2007 and 2010 in Szczecin, Bialystok and Lodz, who were negative for polyps and or cancer and provided blood samples for DNA analysis.

The prostate control group consisted of 3500 cancer-free men aged 23–90 years (mean age: 61.8 years) selected from a part of a population-based survey of 1.5 million residents of West described above.

The first subgroup included 1,026 cancer free males (age range: 23–87 years; mean age: 61.6 years) with no family history of any cancer in a first-degree relative.

These men were selected at random from a registry of patients who participated in the population-based study and were invited for an interview in 2007. Individuals with a first-degree relative diagnosed with cancer were excluded from this control group. The second series of prostate controls consisted of 2474 unselected men at age above 45 (age range: 45–90 years; mean age: 61.9 years). These men were also selected by random from a database of the population-based study and interviewed between 2010 and 2012. During the interview family history of cancer was collected and detailed family history was taken. A blood sample was provided from all men for DNA analyses. Men with a positive family history of prostate cancer were excluded from this group. In total, the control group included cancer-free men age 23–90 years (mean age: 61.8 years).

### Whole exome sequencing

DNA has been isolated using standard methods from peripheral blood leukocytes taken from participants in Department of Genetics and Pathology in Szczecin.

The Illumina Nexter Rapid Capture Expanded Exome kit (target region size = 62 MB, coding exons, UTRs and miRNAs included) was used for capturing sequence target regions. The kit captured 62 Mbp of the human genome covering coding exons in CCDS and RefSeq databases as well as exons annotated by GENCODE project. The captured regions for each sample were barcoded and every two samples were pooled and used for paired-end sequencing for 100 cycles (generating 100 bp reads) on a single lane of Illumina HiSeq2000’s flow-cell.

The 100 million reads for each exome sequence for each individual were aligned to the reference sequence of the human genome using Burrows-Wheeler transform algorithm[14] SAMtools [15] and GATK [16] packages were used for calling variants.

### Sanger sequencing

The new BRCA2 variant identified by whole-exome sequencing in the discovery phase was confirmed by Sanger direct sequencing. The entire coding regions of *BRCA1* (NG_005905) and BRCA2 (NG_012772) were sequenced in 63 amplicons in discovery phase. Sequencing reactions were performed using the BigDye Terminator v3.1 Cycle Sequencing kit (Life Technologies) according to the manufacturer's protocol (dx.doi.org/10.17504/protocols.io.ta7eihn). Sequencing products were analyzed on the ABI Prism 3500XL Genetic Analyzer (Life Technologies). All sequences were compared to the *BRCA1 and BRCA2* RefSeq sequence for variant detection using Mutation Surveyor software (SoftGenetics).

### LOH analysis

Loss of heterozygosity was evaluated in 2 primary tumors from patient carrying germline BRCA2 variant of unknown significance (c.4534 C>T) identified by targeted sequencing.

Using paraffin-embedded tissues we performed microdissection and isolated DNA from primary tumors and healthy tissues for the control. Sanger sequencing of the DNA fragments containing two germline BRCA2 variants of unknown significance identified by WES and targeted sequencing was performed to evaluate potential loss of heterozygosity.

Unstained formalin-fixed, paraffin-embedded (FFPE) slides were microdissected following pathology revision to select tumor and normal areas. DNA from skin FFPE tissues from melanoma patients carrying two identified BRCA2 variants of unknown significance was isolated using the Qiagen DNeasy FFPE Tissue kit.

DNA (50 ng) from normal and tumor areas was amplified, and Sanger sequencing was carried out in parallel. Primers and reaction conditions are available upon request.

### TaqMan genotyping

DNA was isolated from 5 to 10 ml of peripheral blood. The *BRCA2* c.4534 C>T and c.9334G>A variants were genotyped using a TaqMan assay (Applied Biosystems/Life Technologies) and the LightCycler Real-Time PCR 480 system (Roche Life Science) according to the manufacturer's protocol (dx.doi.org/10.17504/protocols.io.ta4eigw). The primer and probe sequences are available upon request. Laboratory technicians were blinded to case-control status. The overall genotyping call rate was 99.3%. The presence of each variant detected by Taqman assay was confirmed by Sanger sequencing as described above.

#### Software prediction of DNA variants

PROVEAN (Protein Variation Effect Analyzer) is a software tool which predicts whether an amino acid substitution or indel has an impact on the biological function of a protein. A delta alignment score is computed for each supporting sequence. The scores are then averaged within and across clusters to generate the final PROVEAN score. If the PROVEAN score is equal to or below a predefined threshold (e.g. -2.5), the protein variant is predicted to have a "deleterious" effect. If the PROVEAN score is above the threshold, the variant is predicted to have a "neutral" effect [17].

SIFT (Sorting Intolerant From Tolerant) uses sequence homology to predict whether an amino acid substitution will affect protein function and hence, potentially alter phenotype. The score is the normalized probability that the amino acid change is tolerated. SIFT predicts substitutions with scores less than 0.05 as deleterious [18].

PolyPhen-2 (Polymorphism Phenotyping v2), available as software and via a Web server, predicts the possible impact of amino acid substitutions on the stability and function of human proteins using structural and comparative evolutionary considerations [19]. The PolyPhen-2 Web interface can be reached at http://genetics.bwh.harvard.edu/pph2/.

MutationTaster is a free web-based application to evaluate DNA sequence variants for their disease-causing potential. The software performs a battery of *in silico* tests to estimate the impact of the variant on the gene product / protein. Tests are made on both, protein and DNA level [20].

In 2008 IARC (International Agency for Research on Cancer) proposed a standardized classification system of five classes of variants based on the degree of likelihood of pathogenicity for application to sequence-based results for cancer predisposition genes (Ref). With the term “pathogenic mutation” we herein mean either class 5 variant (defininitely pathogenic, probability of being pathogenic >0.99) or class 4 variants (likely pathogenic, probability of pathogenic 0.95–0.99) according to IARC classification. With the term variant of unknown significance we mean class 3 variant (uncertain, probability of being pathogenic 0.05–0.949) according to IARC classification [21].

## Results

### Discovery phase

We found neither pathogenic mutations nor variants of unknown significance within *BRCA1* sequence. We found no unequivocal pathogenic mutations in *BRCA2*.

WES identified one *BRCA2* variant of unknown significance: NM_000059.3(BRCA2):c.9334G>A (p.Asp3112Asn, rs759851035).

**Fig 1:**
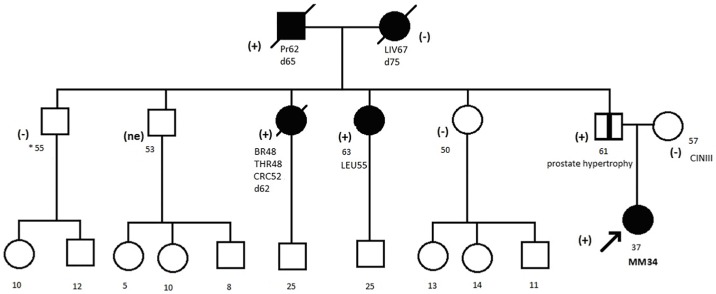
Pedigree of the family with c.9334G>A detected variant. * age. Abbreviation: Pr, prostate cancer; Leu, leukemia; Br, breast cancer; Thr, thyroid cancer; CRC, colorectal cancer; Liv, liver cancer; (+), mutation positive; (-), mutation negative; (ne), not examined.

The variant was found in a female patient diagnosed with MM at the age of 34. Her primary tumor was localized to abdominal skin, Clarks grade III, 0.6mm Breslow thickness, mitotic index 1/mm2, ulceration absent. Evaluation of family history revealed occurrence of cancers of prostate, breast, colon, thyroid and leukemia among family members (Fig 1).

Targeted sequencing of 30 MM cases with cancer familial aggregation identified a second *BRCA2* variant of unknown significance: NM_000059.3(BRCA2):c.4534C>T (p.Arg1512Cys, rs80358684). This variant was found in a female patient diagnosed with synchronous MM at the age of 50 ((https://doi.org/10.7910/DVN/MACR3E).

**Fig 2:**
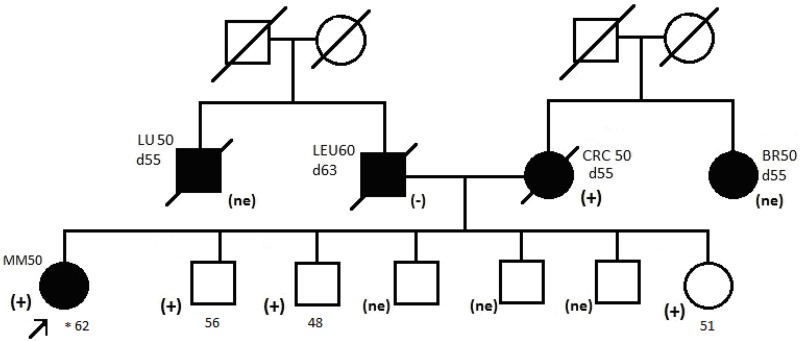
Pedigree of the family with c.4534 C>T detected variant. * age. Abbreviation: Leu, leukemia; Br, breast cancer; Lu, lung cancer; CRC, colorectal cancer; (+), mutation positive; (-), mutation negative; (ne), not examined.

Her first primary tumor was localized on the skin of her lower leg, Clarks grade, 1.4mm Breslow thickness, mitotic index 4/mm2, ulceration absent. Her second primary tumor was localized on the skin of the popliteal fossa, Clarks grade IV, 5.6mm Breslow thickness, mitotic index 1/mm2, ulceration absent. Evaluation of family history revealed occurrence of colorectal cancer affecting the mother of the proband (Fig 2).

### Association study

C.9334G>A change was present in none of the 1230 unselected MM cases and 1696 melanoma controls, none of the 4802 breast cancer cases and 4251 breast controls, none of the 3268 prostate cancer cases and 3478 prostate controls.

C.4534 C>T change was present in none of the 1230 unselected MM cases and 1696 controls. It was present in none of the 4888 breast cases and 4436 breast controls (https://doi.org/10.7910/DVN/MACR3E).

### LOH analysis

We found no loss of heterozygosity of c.4534 C>T in melanoma DNA isolated from the carrier. Due to the absence of tumour tissue available for analysis we were unable to determine if the c.9334G>A variant co-occurred with a second deleterious change.

### Segregation analysis

The c.9334G>A variant segregated with malignancies among the relatives of the proband (Fig 1). Such strong segregation was not observed for the c.4534 C>T variant (Fig 2).

### Software prediction

The c.9334G>A is predilected to affect the BRCA2 protein and thus to be pathogenic according to MutationTaster, Polyphen2 and SIFT algorithms.

The c.4534 C>T variant is considered to be a neutral variant according to MutationTaster, Polyphen2, SIFT and PROVEAN algorithms.

## Discussion

Evidence suggesting possible association between *BRCA1* mutations and melanoma is weak. Deleterious *BRCA1* mutations have been identified in two patients of European origin with primary breast cancer and melanoma [22].

A trend of increasing incidence of melanoma in *BRCA*1 mutation carriers (SIR 3.312, 95% CI 1.511–6.288, p = 0.004) was reported by Mersch et al [12]. All other large familial studies point to no such association [23–26]. The lack of *BRCA1* mutations in our melanoma patients supports the notion that it is not involved in MM development.

In contrast to *BRCA1*, many studies have suggested an association between *BRCA2* mutations and melanoma. Suspected *BRCA2* carriers have shown an increased melanoma risk in the majority (but not all) of reports [27]. As a result of the largest study, the Breast Cancer Linkage Consortium (BCLC) reported an approximate 2.6-fold increase in the risk for CMM among *BRCA2* mutant carrier families [28]. Several studies have evaluated the prevalence of BRCA2 mutations among MM patients.

The *BRCA2* rare nonsense variant (rs11571833) has previously been associated with moderately (OR ranging between 1.26–6.0) increased susceptibility to lung, pancreatic, esophageal and breast cancer [29, 30, 31, 32] was also reported to be significantly over-represented in melanoma patients (OR = 2.80, p = 0.035) [33]. We have previously reported the significantly higher prevalence of the BRCA2 N991D variant in melanoma patients when compared to control subjects (OR = 1.8, p = 0.002), in contrast to T1915M and N372H variants [34]. An association between SNVs in *BRCA2* and melanoma specific overall survival has been suggested by Yin et al [35].

In a recent study of 82 patients with primary melanoma and breast cancers two carriers of *BRCA2* variants were identified: one with a deleterious mutation the second with a variant of unknown significance [15].

In contrast, genotyping of Italian families with an aggregation of melanoma and breast cancer revealed the absence of *BRCA2* mutations, which does not exclude the link between *BRCA2* and melanoma but suggests it is not common [36]. The rarity of founder *BRCA1* and *BRCA2* mutations in Askenazi patients with CMM supports the thesis [37]. Consistently our results point at the rarity of *BRCA2* mutations among Polish melanoma patients. We identified only 2 *BRCA2* variants: c.9334G>A in one out of 96 MM early-onset families and c.4534 C>T in one out of 30 families with aggregation of MM and breast cancer.

BRCA2 mutations are responsible for up to 1% of unselected UK prostate cancer cases [38] and for only up to 0.2% of unselected Polish breast cancer cases [39]. Minor allele frequency of both variants in the Exome Aggregation Consortium (EXAC) population is only 0.00000824. Our results confirm both variants are extremely rare in Polish population as well. The identification of one person within the family harbouring the c.9334G>A variant does, however, lends support to the notion that this specific allele is causative and confers a similar risk to a variety of malignancies.

Both alterations are recognized as variants of unknown clinical significance. We evaluated these variants with algorithms developed to predict the effect of missense changes on protein structure and function such as MutationTaster, Polyphen2, SIFT and PROVEAN. C.9334G>A (rs759851035, Asp3112Asn, POS 32394766) is predicted to be deleterious by three algorithms. The c.9334G>A variant is situated in BRCA2 OB3 domain (oligonucleotide/oligosaccharide-binding, domain 3). The OB3 domain spans between 3052–3186 amino acids, consists of a curved five-stranded beta-sheet that forms a beta-barrel. OB3 domain has a grooves situated between beta 1 and beta 2 sheets and between beta 4 and beta 5 sheets, which allow for strong ssDNA binding) [40].

Although, Asp3112 amino acid is situated in a coil structure between beta 3 and beta 4 sheets and does not bind ssDNA directly, the residue is conserved across mammalian species, suggesting that change might adversely affect protein function. Nevertheless, these predictions have not been yet confirmed by published functional studies.

The prediction that c.9334G>A is deleterious is supported by segregation analysis thereby pointing it towards pathogenicity. The variant was detected in a patient affected with MM, 2 prostate cancer patients, patient with three primary tumours and a female with T-cell leukemia (Fig 1). The spectrum of cancers affecting carriers of c.9334G>A variant is wide- with the exception of leukemia, thyroid and colorectal cancer all other malignancies are part of the BRCA2 phenotype [38]. The most commonly reported cancers with *BRCA*2 mutations include breast, pancreas, prostate, and melanoma [26, 28, 41, 42, 43]. We cannot exclude a possibility of an association between T-cell leukemia and BRCA2 in our patient- in a recent prospective study of 7243 women with a *BRCA1* or a *BRCA2* mutation a higher risk of developing leukemia among women with a *BRCA2* mutation and breast cancer was reported [44].

The c.4534 C>T variant is situated in NH_2_-terminal BRC-repeat (between third and fourth BRC repeat) and does not represent any known functional domain. Although there is a large physico-chemical difference between arginine and cysteine, the arginine residue is weakly conserved, and cysteine amino acid residue is found in multiple mammalian species, suggesting that this missense change does not adversely affect protein function. All four algorithms predict c.4534 C>T (rs80358684, Arg1512Cys, POS 32338889) to be a missense change that does not affect protein function. Genotyping of large cohorts of our melanoma and breast cancer patients and healthy controls revealed that both variants are extremely rare in the Polish population.

We were unable to perform LOH analysis in tumor DNA as it was unavailable from the patient carrying c.9334G>A variant. Nevertheless, the variant segregated with BRCA2-related cancer disease in the family and *in silico* prediction algorithms indicated it affected BRCA2 protein function. It strongly suggests that this BRCA2 variant is pathogenic, and carriers of this change are at an increased risk of BRCA-related cancers, that include breast, prostate and melanoma. In contrast the c.4534 C>T variant did not segregate with cancer and was present in many unaffected individuals. Furthermore, LOH of this variant was not detected. Together, these findings point towards c.4534 C>T being a rare benign polymorphism that should be re-classified as a likely benign variant.

In conclusions, BRCA2 mutations are infrequent among early-onset melanoma patients or MM cases with a familial aggregation with breast cancer. The c.9334G>A variant of *BRCA2*, classified as variant of unknown significance, on current evidence is suggestive of pathogenicity and predisposes to cancers of the breast, prostate and melanoma. Functional studies to describe how the DNA change affects the protein function are needed and due to its rarity in the Caucasian population a large multi-center study to evaluate its penetrance is required.
